# Possible Protective Effect of Sertraline against Cisplatin-Induced Ototoxicity: An Experimental Study

**DOI:** 10.1155/2013/523480

**Published:** 2013-10-01

**Authors:** Murat Ozturk, Selcuk Ucar, Fatih Sarı, Selvet Erdogan, Murat Topdag, Mete Iseri

**Affiliations:** Department of Otolaryngology, Kocaeli University, Faculty of Medicine, 41380 Kocaeli, Turkey

## Abstract

*Background/Objective*. Cisplatin is a widely used chemotherapeutic agent, but its ototoxicity side effect can occur in the majority of patients. Lots of agents were tried to prevent this, but there is not a routine treatment modality yet. The aim of this study was to evaluate the otoprotective effect of sertraline, which is an antidepressant with neuroprotective effects, against cisplatin, in rats. *Design*. Experimental animal study. *Material and Methods*. Forty-eight rats were randomly separated in two groups as groups I and II. Group I was identified as the control group and only a single dose of intraperitoneal cisplatin was administered. In group II, in addition to cisplatin, sertraline was administered to the rats through an oral cannula for ten-day period. Distortion product otoacoustic emission measurements were performed at the first day and the 10th day. *Results*. When the ototoxicity rates after cisplatin in group I and group II in distortion product otoacoustic emission measurements were compared, it was statistically significantly lower in group II in frequencies of 5652, 6165, 6726, 7336, and 7996 Hz (*P* < 0.05). *Conclusion*. Sertraline seems to have a protective effect on cisplatin ototoxicity and could be used to prevent the ototoxicity and also to treat the depression that occurred in cancer patients together.

## 1. Introduction

 Cisplatin (cis-diamminedichloroplatinum) is a widely used chemotherapeutic agent to treat a variety of soft tissue tumors. It is an effective agent especially in head and neck cancers but has various side effects such as nausea, vomiting, neurotoxicity, nephrotoxicity, vestibulotoxicity, and ototoxicity. The most important of these for otolaryngologist is of course ototoxicity, and an elevation in hearing thresholds can occur in up to 75–100% of patients [1]. In experimental studies, cisplatin is characterized with hearing loss in high frequencies (4000–8000 kHz) [2]. Wang et al. showed that giving a dose of 10 mg/kg cisplatin induces apoptosis of cochlear cells, especially in inner and outer hair cells, and stria vascularis [3]. This causes irreversible hearing loss. Patients who are treated with cisplatin are cancer patients already having difficulties in communication, and the hearing loss worsens it. Thence, the incidence of depression increases in these patients. Lots of agents were tried to prevent cisplatin ototoxicity like antioxidant agents, N-acetylcysteine, neurotrophins, p53 inhibitors, and corticosteroids [19]. But lots of these require invasive approaches to deliver the agent into the inner ear and will cause an extra stress to the cancer patients. Sertraline is a selective serotonin reuptake inhibitor used widely in depression treatment. It has neuroprotective and antioxidant effects, stimulates neurogenesis, and increases antiapoptotic protein levels [4, 5]. The aim of this study was to evaluate the otoprotective effect of sertraline, against cisplatin, in rats.

## 2. Material and Methods

### 2.1. Animals

 This study was performed in Kocaeli University Animal Research Laboratory (DETAB) and was approved by the Committee for Ethics in Animal Experiments at Kocaeli University. Forty-eight female, Wistar Albino rats weighing 200 to 250 g and 2 months of age were used. All animals were housed double in standard rat cages in a controlled environment with a temperature of 20°C to 22°C and with 50% to 70% relative humidity, with a 12-hour light-dark cycle. They were fed with rat chow and water.

### 2.2. Experimental Design and Drug Administration

**Figure 1:**
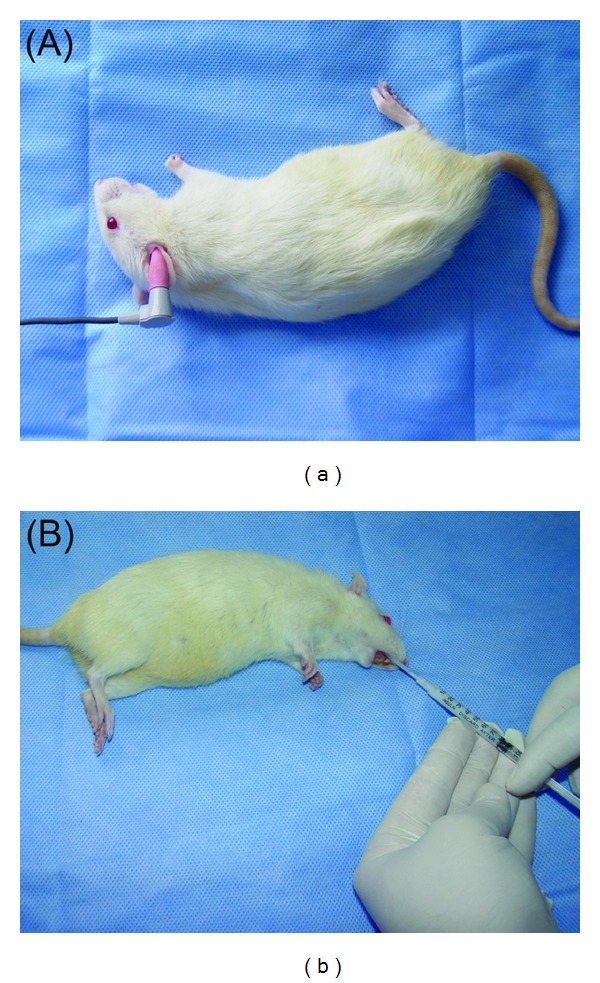
(a) DPOAE measurements with a minimum size rubber tympanometry probe attached to the tip of the probe of the equipment. (b) Administration of sertraline through an oral cannula.

 Forty-eight rats were included in the study. Both ears of all rats were examined by otomicroscope and DPOAE was performed. A total of thirty rats with SPL amplitude in either ears equal or more than 15 dB were deemed eligible for the study. Eighteen rats with SPL amplitude low than 15 dB were excluded from the study. Rats were randomly separated in two groups as groups I and II. Group I was identified as the control groups and only a single dose of intraperitoneal (IP) cisplatin was administered in a dose of 14 mg/kg to the rats in this group. In group II, sertraline diluted with distilled water to 10 mg/mL per dose through an oral cannula at 10 mg/kg/day was administered to the rats, in addition to a single dose of cisplatin at a dose of 14 mg/kg IP (Figure 1). Sertraline treatment began seven days prior to cisplatin administration and was continued for three more days after cisplatin administration; thus, administration continued for a total of ten days. Peroral sertraline and IP cisplatin applications were performed using only ether anesthesia. During DPOAE measurements in all rats, intramuscular 1 mg ketamine and 1 mL xylazine were administered for anesthesia.

### 2.3. Distortion Product Otoacoustic Emission Recordings

 An audiologist and an audiometrist using Otodynamics Ltd. ILOv6 equipment with a minimum size rubber tympanometry probe attached to the tip of the probe of the equipment performed DPOAE measurements. DPOAE (2*f*_1_-*f*_2_ cubic distortion product components) was performed in the General Diagnostic mode, using both DP-gram and input/output (*I/O*) measurements. DPOAEs were measured using stimulations of different frequency and severity. Primer stimulant severity was equalized to 65 dB in DP-gram measurements (*L*_1_ = *L*_2_). The two different frequencies (*f*_1_ and *f*_2_) were arranged as *f*_2_/*f*_1_, so that the most powerful responses would be obtained. DP-gram measurements were performed in 1001, 1501, 2002, 3003, 4004, 4358, 4761, 5188, 5652, 6154, 6726, 7336, and 7996 Hz *f*_2_ frequencies. When *I/O* measurements were performed, responses in situations such as *f*_1_ = *f*_2_ = 80, 70, and 65/55 and in gradually decreasing severity were recorded. Both measurements of threshold and over threshold of *I/O* functions were performed using primary sound tones decreasing from 80 dB to 50 dB in 5 dB decrements. Measurements were performed at 1001, 1501, 2002, 3003, 4004, 4358, 4761, 5188, 5652, 6154, 6726, 7336, and 7996 Hz frequencies. 

 Noise levels for both DP-gram and *I/O* functions were measured at frequencies more than 50 Hz. The OAEs equal to 3 dB or more than the noise level in 2*f*_1_-*f*_2_ frequencies were accepted as hearing is present during the measurements. If the OAE is less than 3 dB, it is accepted as hearing is absent which means that ototoxicity occurred in this ear. Responses obtained during the first round are recorded in all measurements. In group I and group II, *I/O* measurements were performed in each frequency for situations such as *f*_1_ = *f*_2_ = 80, 70, and 65/55 in 1001, 1501, 2002, 3003, 4004, 4358, 4761, 5188, 5652, 6165, 6726, 7336, and 7996 Hz prior to drug administration. Oral sertraline was given to group II for 7 days in a dose of 10 mg/kg/day. A single dose of IP cisplatin was given in both group I and group II in a dose of 14 mg/kg on day seven. Three days after cisplatin application, *I/O* measurements in *f*_1_ = *f*_2_ = 80, 70, and 65/55 for each frequencies which were 1001, 1501, 2002, 3003, 4004, 4358, 4761, 5188, 5652, 6165, 6726, 7336, and 7996 Hz were performed and recorded in surviving rats in group I and group II. 

### 2.4. Statistical Analysis

 The statistical analysis was done by Kocaeli University Public Health Department. DPOAE amplitudes were analyzed by Mann-Whitney test, and the presence of ototoxicity is analyzed by Chi-square test. Data were analyzed by using SPSS for Windows 13.0.

## 3. Results

 Seven out of fourteen rats died three days after cisplatin administration in group I. Two of the sixteen rats in group II died due to ether inhalation during sertraline administration (before cisplatin administration), and additional two died for reasons other than ether inhalation (on the 2nd day of cisplatin administration). On the 3rd day of cisplatin administration, *I/O* measurements in *f*_1_ = *f*_2_ = 80, 70, and 65/55 in each of the frequencies of 1001, 1501, 2002, 3003, 4004, 4358, 4761, 5188, 5652, 6165, 6726, 7336, and 7996 Hz were performed and recorded in the seven surviving rats in group I (14 ears) and in twelve surviving rats in group II (24 ears) and DPOAEs were performed.

**Figure 2:**
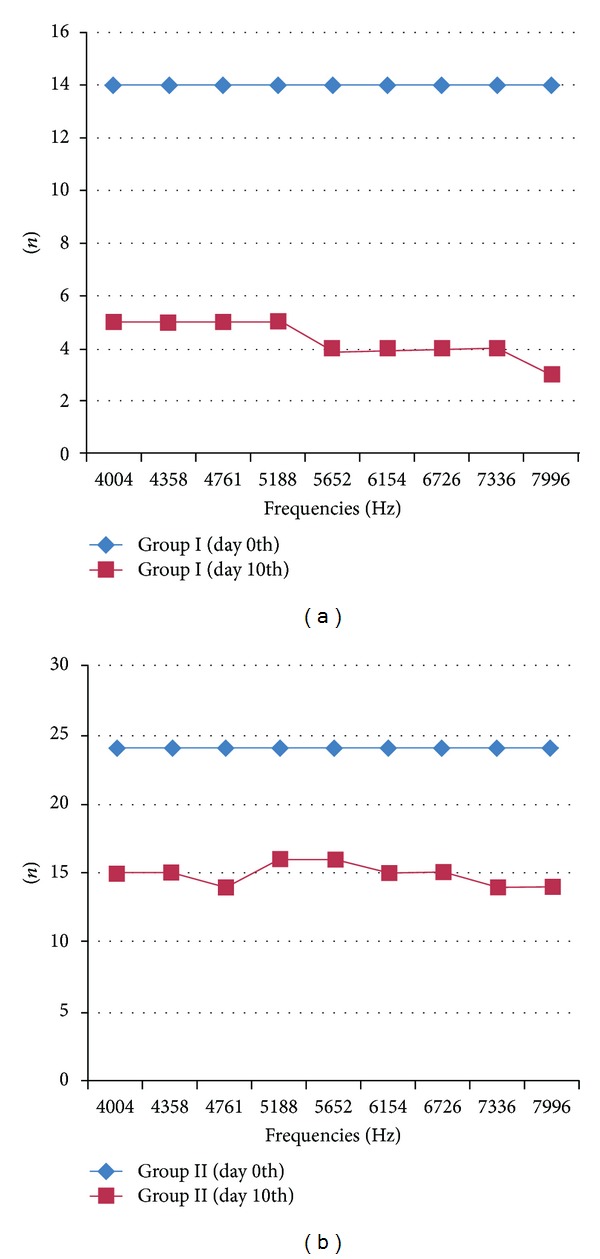
The number of the rats that have hearing before and after cisplatin administration in group I (a) and in group II (b).

**Figure 3:**
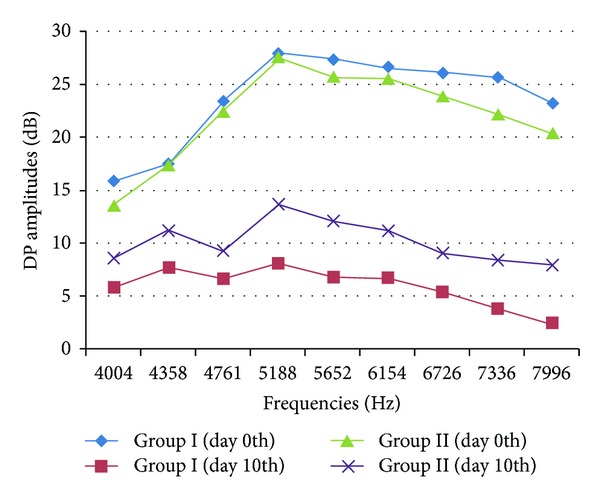
*I/O* measurement at *f*_1_/*f*_2_ = 65/55 in group I and group II, DP graph before and after cisplatin administration.

 The numbers of hearing ears with frequencies before and after cisplatin administration in groups I and II are given in Figure 2. The DPOAE amplitude averages of all groups with frequencies are given in Figure 3. When we look at Figure 3, it looks like there was a difference between groups 1 and 2. But; when we compare them statically with Mann-Whitney test, there were no significant differences between them.

 In fourteen ears (seven rats) in group I in *I/O* measurements for *f*_1_/*f*_2_ = 65/55 after cisplatin administration, ototoxicity was observed in nine (64%) at frequencies of 4004, 4358, 4761, and 5188 Hz, 10 (71%) at frequencies of 5652, 6165, 6726, and 7336 Hz, and eleven (78%) at a frequency of 7996 Hz. In 24 ears (twelve rats) in group II, in *I/O* measurements for *f*_1_/*f*_2_ = 65/55 after cisplatin administration, ototoxicity was observed in eight (33%) at frequencies of 5188 and 5652 Hz, in nine (37%) at frequencies of 4004, 4358, 6165, and 6726, and in ten (41%) at frequencies of 4761, 7336, and 7996 Hz. 

**Figure 4:**
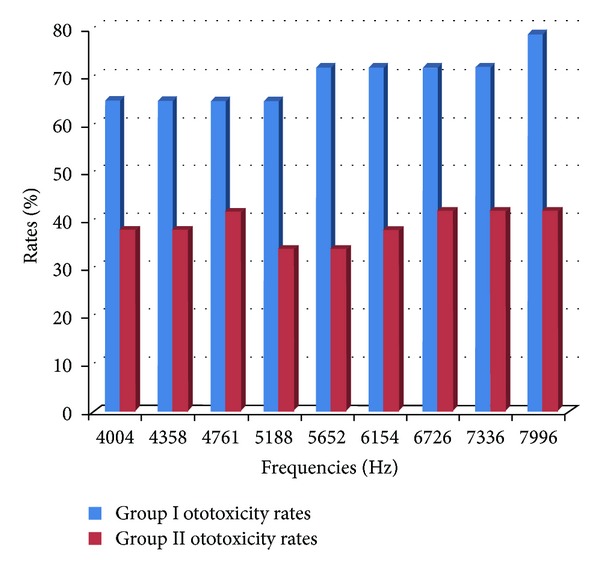
The occurrence of ototoxicity rates at the 10th day after cisplatin administration in groups I and II.

 When the ototoxicity rates after cisplatin in group I and group II in *I/O* measurements in *f*_1_/*f*_2_ = 65/55 were compared, it was statistically significantly lower in group II in frequencies of 5652, 6165, 6726, 7336, and 7996 Hz (*P* < 0,05). No significant differences were observed in lower frequencies such as 1001, 1501, 2002, 3003, 4004, 4358, 4761, and 5188 Hz. The ototoxicity rates of two groups at the 10th day are given in Figure 4.

## 4. Discussion

 There are lots of chemotherapeutic agents nowadays which have less side effects but cisplatin is still more effective than most of these agents, and so it is still being used widely. For this reason, ear nose and throat specialists frequently encounter adverse effects of cisplatin such as vestibulotoxicity and ototoxicity. Risk increases, especially in younger patients, with large cumulative doses, individuals with prior hearing loss, renal disease, or with a history of radiation to brain or skull base [6].

 Cisplatin is known to produce ototoxicity through some mechanisms such as myelin sheath separation in type 1 spiral ganglion cells, apoptosis induction in the organ of Corti, increased free oxygen radicals in cochlear cells. In addition, it has deleterious effects on the basal turn stria vascularis, including strial edema, bulging, rupture, and compression of the marginal cells, and depletion of organelles from the cytoplasm. Molecules preventing oxidative stress are glutathione and the antioxidant enzymes, heat shock proteins, adenosine A1 receptors, NRF2 and heme-oxygenase-1, the kidney injury molecule (KIM-1), and several thiol antioxidants. In addition, intratympanic dexamethasone application has also been shown to be preventive against cisplatin toxicity [6]. Perilymphatic perfusion of sodium thiosulfate in guinea pigs prevents cisplatin ototoxicity [7], whereas application to the round window membrane using an osmotic mini pump is not effective in preventing cisplatin ototoxicity [8]. N-Acetylcysteine protects against cisplatin ototoxicity whether it is administered systemically or transtympanically [6, 9, 10]. Amifostine was found to protect against peripheral ototoxicity in the hamster but also to increase neurotoxicity [11]. Other antioxidant agents D-methionine, alpha-tocopherol, aminoguanidine, sodium salicylate, and ebselen were also found to prevent ototoxicity of cisplatin [6, 12, 13]. A1 adenosine receptor agonist, R-PIA [14], neurotrophins such as neurotrophin-3 [15], flunarizine [16], intracochlear perfusion of inhibitors of caspase-3 and caspase-9 [3], XIAP (the X-linked inhibitor of apoptosis protein) [17], and the p53 inhibitor pifithrin-alpha [18] were also found as protective. In addition, intratympanic dexamethasone application has also been shown to be preventive against cisplatin toxicity [19]. But lots of these studies are in vitro studies, and investigators used invasive approaches to deliver the agent into the inner ear [6].

 Sertraline is a selective serotonin reuptake inhibitor (SSRI) and is widely used for the treatment of patients with depression and severe anxiety disorders. It is also shown that SSRIs can also stimulate neurogenesis and protect neurons against metabolic/oxidative insults [4, 5]. Duan et al. studied sertraline and found that sertraline increases levels of brain-derived neurotrophic factor levels, preserves chaperone protein HSP70 levels and antiapoptotic protein Bcl-2 levels, restores depleted serotonin levels, retards motor behavioral impairment, and enhances neurogenesis [5]. Duan and Kumar stated enhancing effect of sertraline on neurogenesis and its antioxidant effect [5, 20].

 This study especially takes into account the antioxidant, neuroprotective effects of sertraline and protective effects of antiapoptotic protein Bcl-2. The probable preventive effect of sertraline in cisplatin ototoxicity was evaluated in this study. No statistically significant differences were observed between the cisplatin group and cisplatin + sertraline group in frequencies lower than 5000 Hz while in frequencies higher than 5000 Hz, the cisplatin + sertraline group had statistically significantly better results in hearing. Sertraline in a dose of 10 mg/kg/day was administered, beginning seven days prior to cisplatin treatment and ending three days after cisplatin administration, for a total of ten days. Future studies with higher doses in longer durations might result in more protective effects or similar results might be obtained with lower doses and shorter durations. Particularly if lower doses yield successful results, a suggestion could be made for clinical practice such as administering low dose sertraline to patients who are taking cisplatin with risk factors for toxicity.

 Some invasive methods such as intratympanic steroid application have also been demonstrated to be effective in preventing ototoxicity. However, it is difficult to apply these methods in patients already receiving a lot of invasive or noninvasive treatments because of their malign diseases. These additional procedures might cause additional anxiety for the patient that would result in compliance problems in clinical practices. Therefore, using easily tolerated and orally administered agents with no adverse effects, such as sertraline, would be advantageous.

 It is not practical to suggest sertraline use in all patients using cisplatin to prevent ototoxicity. However, if the hearing and communication are important tasks from the perspective of the patients with risk factors, its use can be suggested under guidance of future clinical studies. In addition, if depression is present in patients taking cisplatin and medical treatment is needed for depression, sertraline could be the drug of choice because of its decreasing effect of ototoxicity. The authors of this study plan to run a clinical study to compare the hearing thresholds of patients taking cisplatin and sertraline for their depression with a control group.

## 5. Conclusion 

 Cisplatin has been widely used in spite of its ototoxicity, which is important for the specialty of ear, nose, and throat, and all other adverse effects. When the increase in the number of new cancer cases and quality of life are taken into account, agents that would decrease the ototoxic effects of drugs like cisplatin should be evaluated and used. For this reason, sertraline was used in this study, and it was determined that sertraline would be beneficial to treat depression, could improve communication problems, and also could decrease hearing loss due to ototoxicity. Future clinical and experimental studies would be enlightening.
